# *Cysteine Proteinase-1* and Cut Protein Isoform Control Dendritic Innervation of Two Distinct Sensory Fields by a Single Neuron

**DOI:** 10.1016/j.celrep.2014.02.003

**Published:** 2014-02-27

**Authors:** Gray R. Lyons, Ryan O. Andersen, Khadar Abdi, Won-Seok Song, Chay T. Kuo

**Affiliations:** 1 Department of Cell Biology, Duke University School of Medicine, Durham, NC 27710, USA; 2 Medical Scientist Training Program, Duke University School of Medicine, Durham, NC 27710, USA; 3 Brumley Neonatal-Perinatal Research Institute, Department of Pediatrics, Duke University School of Medicine, Durham, NC 27710, USA; 4 Department of Neurobiology, Duke University School of Medicine, Durham, NC 27710, USA; 5 Preston Robert Tisch Brain Tumor Center, Duke University School of Medicine, Durham, NC 27710, USA; 6 Duke Institute for Brain Sciences, Duke University School of Medicine, Durham, NC 27710, USA

## Abstract

Dendrites often exhibit structural changes in response to local inputs. Although mechanisms that pattern and maintain dendritic arbors are becoming clearer, processes regulating regrowth, during context-dependent plasticity or after injury, remain poorly understood. We found that a class of *Drosophila* sensory neurons, through complete pruning and regeneration, can elaborate two distinct dendritic trees, innervating independent sensory fields. An expression screen identified *Cysteome proteinase-1* (*Cp1*) as a critical regulator of this process. Unlike known ecdysone effectors, *Cp1*-mutant ddaC neurons pruned larval dendrites normally but failed to regrow adult dendrites. Cp1 expression was upregulated/concentrated in the nucleus during metamorphosis, controlling production of a truncated Cut homeodomain transcription factor. This truncated Cut, but not the full-length protein, allowed *Cp1*-mutant ddaC neurons to regenerate higher-order adult dendrites. These results identify a molecular pathway needed for dendrite regrowth after pruning, which allows the same neuron to innervate distinct sensory fields.

## INTRODUCTION

Dendrites are the primary sites of information input for neurons. Their initiation, arborization, targeting, and function are regulated by a series of finely tuned cellular events (Jan and Jan, 2010; Stuart et al., 2008). Critical for the proper wiring of neural circuits, defects in dendrite development and function have been linked to human neurodevelopmental and psychiatric diseases, including autism, fragile X syndrome, and schizophrenia (Kulkarni and Firestein, 2012; Penzes et al., 2011). Dendrites can also remodel after their initial arborization. This process is often coupled to neuronal activity inputs from external stimuli (Chen and Nedivi, 2010; Tavosanis, 2012) and presents a potential cellular basis for sensory map remodeling (Feldman and Brecht, 2005; Hickmott and Steen, 2005). Although the molecular mechanisms that pattern and maintain the proper dendritic tree/field are becoming clearer, the processes regulating dendritic rewiring remain poorly understood.

*Drosophila* peripheral nervous system dendritic arborization (da) neurons, classified into four classes (I–IV) based on their location and dendritic arbor complexity, have served as a powerful model system for studying conserved pathways controlling dendrite morphogenesis (Parrish et al., 2007). We showed previously that class IV da (C4 da) neurons undergo ecdysone hormone-induced pruning and subsequent regrowth of dendritic arbors during metamorphosis (Kuo et al., 2005). This remodeling is initiated by intracellular events downstream of nuclear hormone receptor signaling (Kanamori et al., 2013; Kirilly et al., 2009; Kuo et al., 2005, 2006; Lee et al., 2009; Williams et al., 2006) and extracellular events controlled by phagocytes (Williams and Truman, 2005) and epidermis (Han et al., 2014). After pruning, C4 da neurons regrow dendrites that innervate the adult sensory fields (Kuo et al., 2005), but the mechanisms controlling this dendrite regrowth remain largely unknown.

Here, we show that ddaC C4 da neurons regenerate adult dendritic arbors in a different manner after pruning than initially during development. Starting with an expression screen, we identified *Cysteome proteinase-1* (*Cp1*) and its critical role in regulating ddaC neuron dendrite regeneration to innervate the adult sensory fields.

## RESULTS AND DISCUSSION

### Regrowth of ddaC Sensory Neuron Dendrites

**Figure 1:**
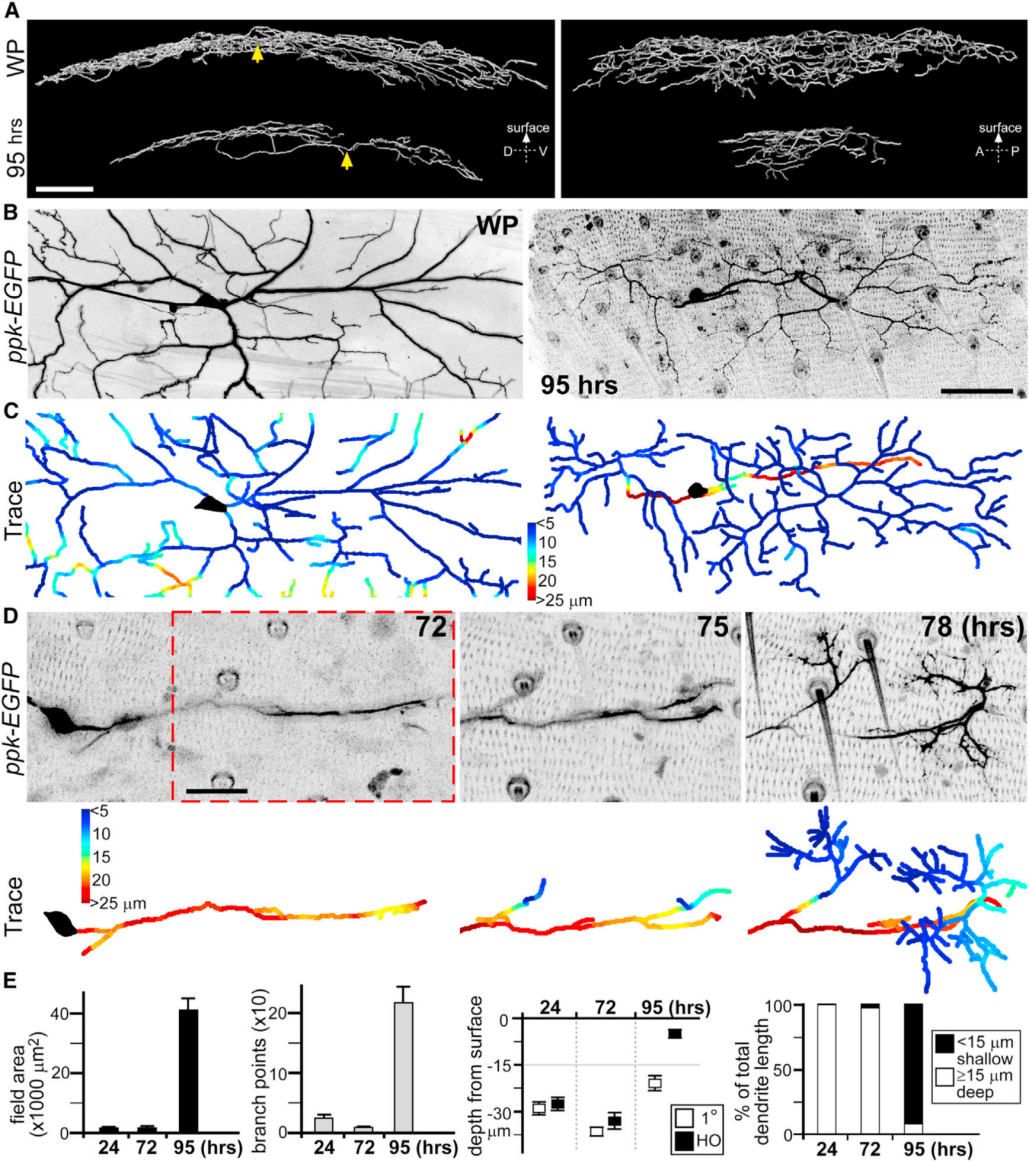
ddaC Neuron Dendrite Regrowth after Pruning (A) Reconstruction of ddaC neuron dendrites at the start of metamorphosis prior to pruning (white pupae [WP]), and just before eclosion after dendrite regrowth (95 hr APF). Arrows point to soma positions. D, dorsal; V, ventral; A, anterior; P, posterior. Scale bar, 50 μm. See Movie S1. (B) x-y view maximum projections from live imaging of abdominal segment ddaC neuron at WP and 95 hr APF (using *ppk-EGFP* reporter). Scale bar, 50 μm. (C) Corresponding depths of dendritic arbors from the body wall are colorimetrically represented in traces below: shallow dendrites are shaded blue; deep dendrites are shaded red. (D) Representative time-lapse live imaging of ddaC neuron secondary dendrite growth from primary dendrite, during regrowth at 72, 75, and 78 hr APF. Depth of arbors from the body wall is colorimetrically represented in corresponding traces below. Seventy-five and 78 hr APF images represent same field of view as in red-dashed box. Scale bar, 25 μm. See Movie S3. (E) Quantitative analyses of ddaC neuron dendritic arbor changes during regrowth: field area, branchpoints, depth of primary (1°) and higher-order (HO) dendrites from surface, and percentages of total dendrite length that are shallow (within 15 μm from body wall) or deep (≥15 μm) (n = 8 in all groups. Error bars represent SEM. Images of ddaC neuron dendrites are inverted to black on white for clarity. Anterior is up and dorsal is right in all images. See also Figures S1 and S2.

It is likely that many of the developmental pathways used to elaborate larval sensory neuron dendrites will be reused during regrowth. We reasoned that if mirrored programs were used, then the regrown dendritic trees should morphologically resemble earlier larval shapes. The ddaC C4 da neurons maintain a stereotyped 2D dendritic morphology prior to metamorphosis (Han et al., 2012; Kim et al., 2012). This is established first by inserting early dendrites into the body wall during development, followed by dendritic growth that is scaled to concurrent expansion of the larval body wall and receptive fields (Parrish et al., 2009). Using live imaging of *pickpocket* (*ppk*)-*EGFP* reporter line to follow the abdominal segment ddaC neurons through metamorphosis, we found that their dendritic arbors changed into a different architecture after regrowth (Figure 1A; Movie S1). In addition to covering a smaller field, the soma and primary dendrites reside in a separate, deeper plane than higher-order dendritic branches that project to the body wall above (Figures 1A and S1A; Movie S1). To quantify the changes (Figure 1B), we developed a software script to track the depth of dendrites from the body wall and represented this distance colorimetrically (deeper arbors in red, shallower in blue, Figures 1C and S1B).

To understand the steps necessary to elaborate this dendritic tree after pruning, we performed time-lapse imaging of ddaC neurons during metamorphosis. Shortly after complete dendrite pruning at 24 hr after puparium formation (APF), ddaC neurons initiated dendrite regrowth, projecting primary dendrites along the wall from a lateral-to-medial direction (Figure S2A). This initial phase of dendritic growth was highly dynamic, with numerous neurite extensions/retractions (Figure S2A; Movie S2). Most of these neurites are transient structures because the primary dendrites continued to elongate without much elaboration of higher-order branches (Figures 1D and S2B). At later stages, between 60 and 72 hr APF, we observed the first stabilization of secondary dendrites branching from the primary dendrites toward the body wall above (Figure 1D). These secondary dendrites did not branch further until they reached the body wall, at which time there was a rapid expansion of higher-order dendritic branches close to the body wall (Figure 1D; Movie S3). This late expansion accounted for the majority of mature ddaC neuron dendritic field coverage at 95 hr APF just before eclosion (Figure S2C). Although initiation of the primary dendrite after pruning is rather stereotyped, the subsequent targeting/expansion of higher-order dendrites at the body wall differed between neighboring ddaC neurons. Quantification showed the temporal relationships of this process (Figure 1E), representing a different approach from receptive field scaling used by these same ddaC neurons during larval dendrite growth (Parrish et al., 2009).

### Identification of *Cp1* Regulating ddaC Neuron Dendrite Regrowth

**Figure 2:**
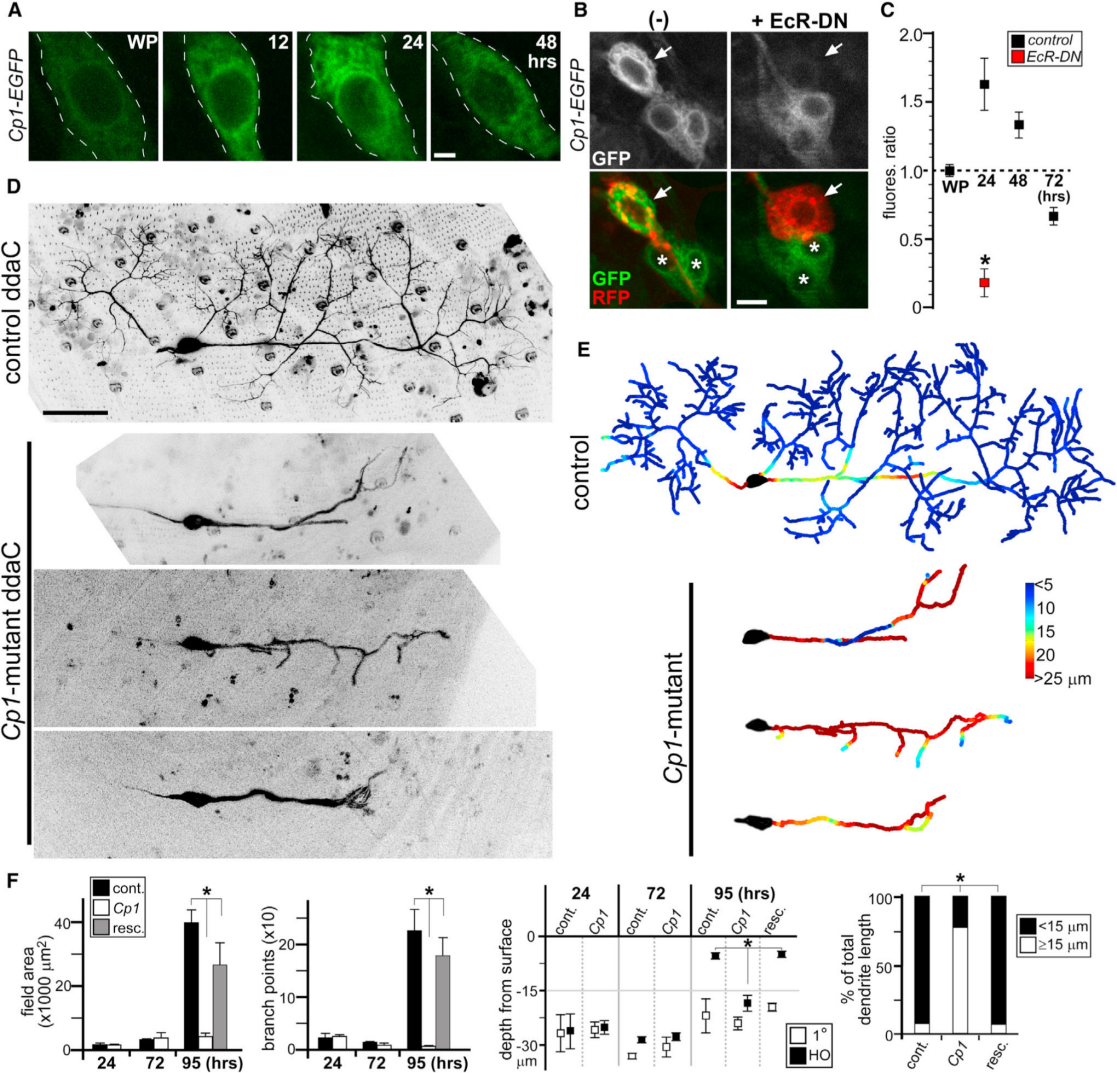
Cp1 Function during Dendrite Regrowth (A) Live imaging of *Cp1-EGFP* fluorescence in ddaC neurons during metamorphosis. (B) Live imaging of *Cp1-EGFP* fluorescence in ddaC neurons expressing EcR-DN receptor (*ppk-Gal4*; *UAS-mCD8::RFP*; *UAS-EcR-DN*). Arrows point to RFP^+^ ddaC neurons. Note that *Cp1-EGFP* expression in neighboring cells (*) is unaffected by EcR-DN expression via *ppk-Gal4* driver. (C) Quantitative analyses of *Cp1-EGFP* fluorescence levels: average ratio of EGFP/RFP signal from ddaC neurons in WP is set to 1 (n = 6 in all groups). *p < 0.005, Wilcoxon two-sample test. Error bars represent SEM. (D) Live imaging of control and *Cp1*-mutant ddaC neuron clones at 95 hr APF. Three representative *Cp1*-mutants are shown. (E) Corresponding colorimetric representation of dendritic arbor depths in (D). (F) Quantitative analyses of *Cp1*-mutant dendrite regrowth defects: field area, branchpoints, depth of primary (1°) and higher-order dendrites from surface, and percentages of total dendrite length at 95 hr APF that are shallow (within 15 μm from the body wall) or deep (≥15 μm). cont., control; resc., rescue. n ≥ 6 in all groups. *p < 0.001, one-way ANOVA. Error bars represent SEM. Scale bars, 2 mm (A), 5 mm (B), and 50 μm (D). See also Figure S3.

We hypothesized that if variations in molecular programs are needed to grow two different sets of dendrites in the same neuron, then the genes involved will likely change their expression levels in a context-dependent manner. We set out to identify such genes in ddaC neurons during dendrite regrowth. An expression screen of the EGFP-FlyTrap collection identified stock ZCL2854, corresponding to EGFP insertion into the *Cp1* gene, that showed increased EGFP expression during ddaC neuron dendrite remodeling (Figure 2A). We quantified *Cp1-EGFP* fluorescence levels in ddaC neurons by normalizing EGFP intensity to internal *UAS-mCD8::RFP* fluorescence driven by *ppk-Gal4*, which remained relatively constant throughout (Figure 2C; data not shown). We showed previously that dendrite remodeling in these neurons is initiated by nuclear hormone receptor signaling (Kuo et al., 2005). To confirm that the increase in *Cp1-EGFP* expression during metamorphosis is controlled by the *Drosophila* hormone ecdysone, we blocked ecdysone signaling by expressing a dominant-negative ecdysone receptor (*UAS-EcR-DN*) in ddaC neurons (Kirilly et al., 2009; Kuo et al., 2005). This effectively attenuated *Cp1-EGFP* upregulation during metamorphosis (Figures 2B and 2C).

Cp1 contains an evolutionarily conserved cysteine proteinase domain (Tryselius and Hultmark, 1997), but its function in *Drosophila* is poorly understood, with no previous link to neuronal development/function. Because in our hands *Cp1*-mutants were lethal (both *Cp1^llcnbw38^* and *Cp1^c03987^* alleles), we generated *Cp1*-mutant ddaC neuron clones. Unlike other signals downstream of ecdysone identified thus far during ddaC neuron dendrite remodeling (Kanamori et al., 2013; Kirilly et al., 2009), *Cp1*-mutant ddaC neurons pruned their larval dendrites normally during metamorphosis, followed by extension of their primary dendrites similar to controls (Figures S3A and S3B). But they failed to properly elaborate higher-order dendrites during the expansion phase when these dendrites target the body wall (Figures 2D–2F and S3B). These defects can be partially recovered by reexpressing Cp1 in mutant clones using a *UAS-Cp1* trans-gene (Figure S3D). In contrast to ecdysone control of endogenous Cp1 expression (Figure 2B), this Cp1 re-expression is under Gal4/UAS control; thus, it may not fully recover wild-type dendritic morphologies. We did not observe obvious dendritic morphology defects in larval ddaC neurons expressing Cp1 via *UAS-Cp1* transgene (Figure S3C; data not shown).

### Cut Transcription Factor Isoform Regulates ddaC Neuron Dendrite Regrowth

**Figure 3:**
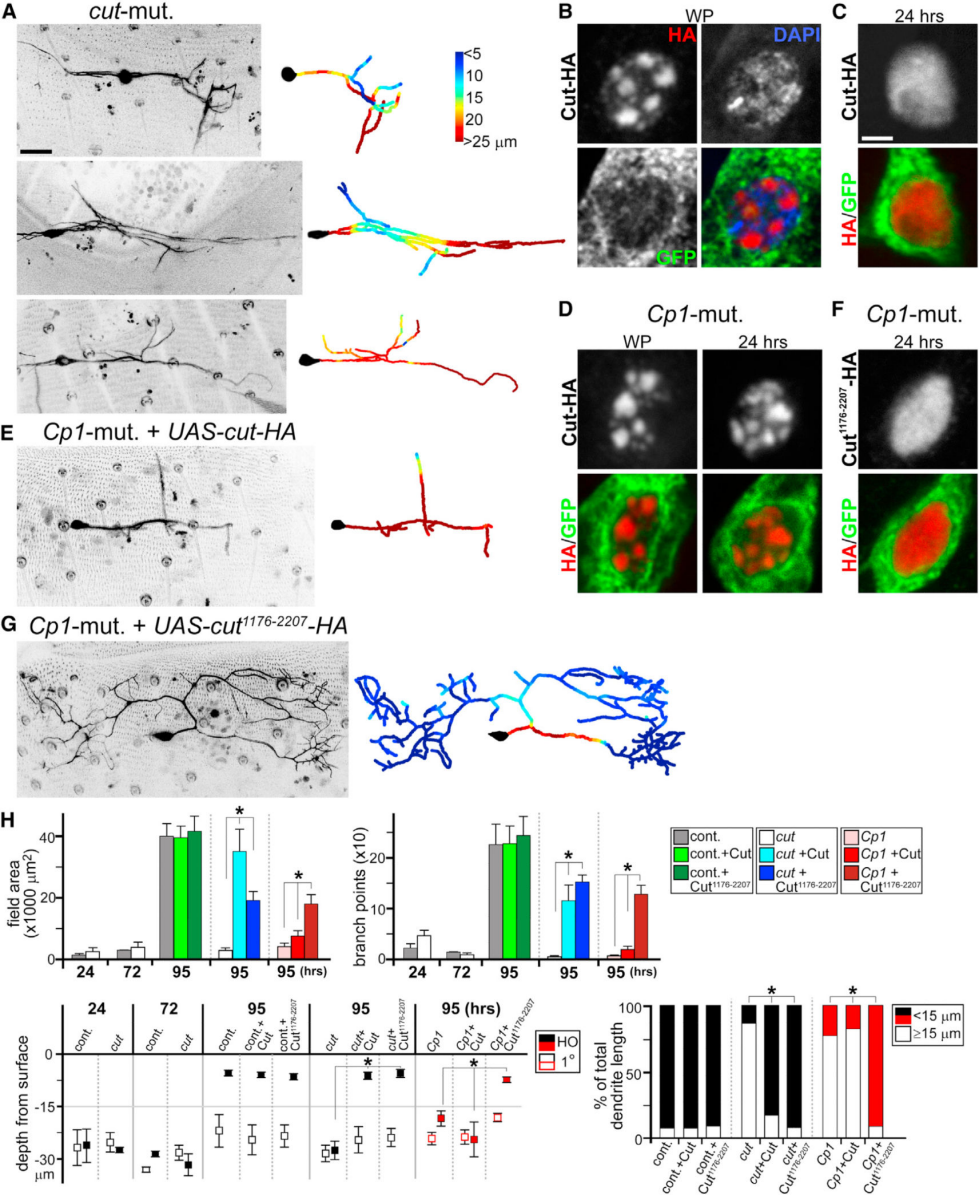
Cp1-Dependent Cut Isoform Production Required for Dendrite Regrowth (A, E, and G) Live imaging of ddaC neuron clones at 95 hr APF, with colorimetric representations of dendritic arbor depth in right panels. (A) Representative *cut*-mutant ddaC clones are shown. (E) *Cp1*-mutant ddaC neuron expressing full-length Cut is shown. (G) *Cp1*-mutant ddaC neuron expressing truncated Cut^1176-2207^ is shown. (B–D and F) GFP and HA IHC antibody staining of ddaC neurons (*ppk-Gal4; UAS-mCD8::GFP; UAS-cut-HA* or *UAS-cut^1176-2207^-HA*) during metamorphosis. (B and C) Cut-HA nuclear localization patterns in control background at WP (B) and 24 hr APF (C) are shown. (D) Cut-HA nuclear patterns in *Cp1*-mutant clones at WP and 24 hr APF are shown. (F) Cut*1176-2207*-HA nuclear patterns in *Cp1*-mutant clones at 24 hr APF are shown. (H) Quantitative analyses of *cut*-mutant dendrite regrowth defects, *cut*-mutant rescue experiments with full-length Cut or Cut^1176-2207^, and Cp1/Cut rescue experiments: field area, branchpoints, depth of primary (1°) and higher-order dendrites from surface, and percentages of total dendrite length at 95 hr APF that are shallow (within 15 μm from the body wall) or deep (≥15 μm) (n ≥ 5 in all groups). *p < 0.001, one-way ANOVA. Error bars represent SEM. Scale bars, 25 mm (A, E, and G) and 2 mm (B–D and F). See also Figures S3 and S4.

Because *Cp1* function in *Drosophila* is unclear, we took a candidate approach to understand its regulation of ddaC neuron dendrite regrowth. One of the reported protein targets for cathepsin L (Ctsl), the mammalian homolog to Cp1, is homeodomain transcription factor Cut-like 1 (Cux1) (Goulet et al., 2004). During cell-cycle progression, Ctsl cleaves Cux1 between the first and second Cut repeats, generating a truncated protein containing the second and third Cut repeats and homeodomain, with different transcriptional properties to the full-length protein (Goulet et al., 2004; Moon et al., 2001). Cux1 is related to Cut, a key determinant of *Drosophila* peripheral sensory neuron dendrite arborization during development (Grueber et al., 2003). We asked whether Cut is part of the Cp1 pathway regulating ddaC neuron dendrite regrowth by first generating *cut*-mutant ddaC neuron clones. Consistent with earlier reported defects in larvae (Grueber et al., 2003), these neurons exhibited altered dendrites at the white pupae stage, but the dendrites pruned normally during metamorphosis, followed by regrowth of primary dendrites from the soma (data not shown). Thereafter, *cut*-mutant ddaC neurons showed a severe defect in arborization of higher-order dendritic branches targeting the body wall (Figure 3A). Quantification of multiple clones, followed continuously from identification at the start of metamorphosis to just prior to eclosion, showed the severity of their dendrite regrowth defects (Figures 3A and 3H; data not shown). These defects can be partially recovered by reexpressing Cut in mutant clones using a *UAS-cut* transgene (Figures S3E and 3H).

The similarities in higher-order dendrite defects between *Cp1* and *cut*-mutant ddaC neurons led us to probe further their molecular connections. We tested whether a portion of Cp1's function regulating ddaC neuron dendrite regrowth is to produce a truncated Cut isoform. For this, we inserted a 3’ HA tag to better resolve immunohistochemical (IHC) staining signals during nuclear localization and generated a *UAS-cut-HA* transgenic line. In vivo functionality of this tagged transgene was confirmed by successful repeat of rescue experiment in *cut-*mutant ddaC neurons (as in Figure S3E; data not shown). We next crossed this line to *ppk-Gal4*; *UAS-mCD8::EGFP* trans-genes to visualize HA-tagged Cut protein in EGFP^+^ ddaC neurons during metamorphosis. IHC staining showed that Cut-HA is localized to nuclear “spots” in ddaC neurons in white pupae at the start of metamorphosis (Figure 3B). These spots did not appear to colocalize with DAPI spots corresponding to heterochromatin (Figure 3B). Interestingly, at 24 hr APF, HA antibody staining showed a change in nuclear localization from punctate to generally diffuse patterns, excluding the nucleolus (Figure 3C; data not shown). If the punctate Cut-HA localization was caused by increased expression alone, then we would expect to detect punctate patterns in white pupae and at 24 hr APF. By contrast, if these changes reflect binding specificities of full-length and putative truncated Cut isoforms, then one would predict a defect in this transition in the absence of Cp1. When *UAS-cut-HA* was expressed in *Cp1*-mutant ddaC neurons, HA antibody staining showed distinct nuclear spots in both white pupae and at 24 hr APF (Figure 3D). Moreover, this Cut-HA expression in *Cp1*-mutant ddaC neurons did not recover dendrite regrowth defects (Figures 3E and 3H).

Next, we hypothesized that if the diffuse nuclear staining pattern of Cut-HA at 24 hr APF corresponded in part to Cp1-dependent production of a truncated Cut isoform, then this isoform should display similarly diffuse localization patterns independent of Cp1. Based on homology to mammalian Cux1 cleavage by Ctsl (Moon et al., 2001), we generated a *UAS-cut^1176-2207^-HA* transgenic line containing amino acids 1,176–2,207 from the full-length 2,207 amino acid Cut protein (including second and third Cut repeats and homeodomain). When expressed in *Cp1*-mutant ddaC clones, truncated Cut^1176-2207^-HA showed consistently diffuse nuclear localization at 24 hr APF (Figure 3F). Furthermore, whereas the full-length Cut-HA did not alter the *Cp1*-mutant phenotype (Figures 3E and 3H), this truncated Cut^1176-2207^-HA allowed the regrowth of higher-order dendrites in *Cp1*-mutant ddaC neurons (Figures 3G and 3H). Consistent with these results, Cut^1176-2207^-HA was able to promote regrowth of higher-order dendrites in *cut*-mutant ddaC neurons (Figures 3H and S3F). This partial recovery in *Cp1*-mutant ddaC neurons by Cut^1176-2207^-HA was specific because other Cut truncations containing differing domains failed to do so (Figure S4A). To ensure relative levels of protein expression, all *UAS-rescue* Cut constructs used were similarly knocked into the attP2 locus.

### Cut Isoform Induces ddaC Neuron Dendrite Defects in Third-Instar Larvae

**Figure 4:**
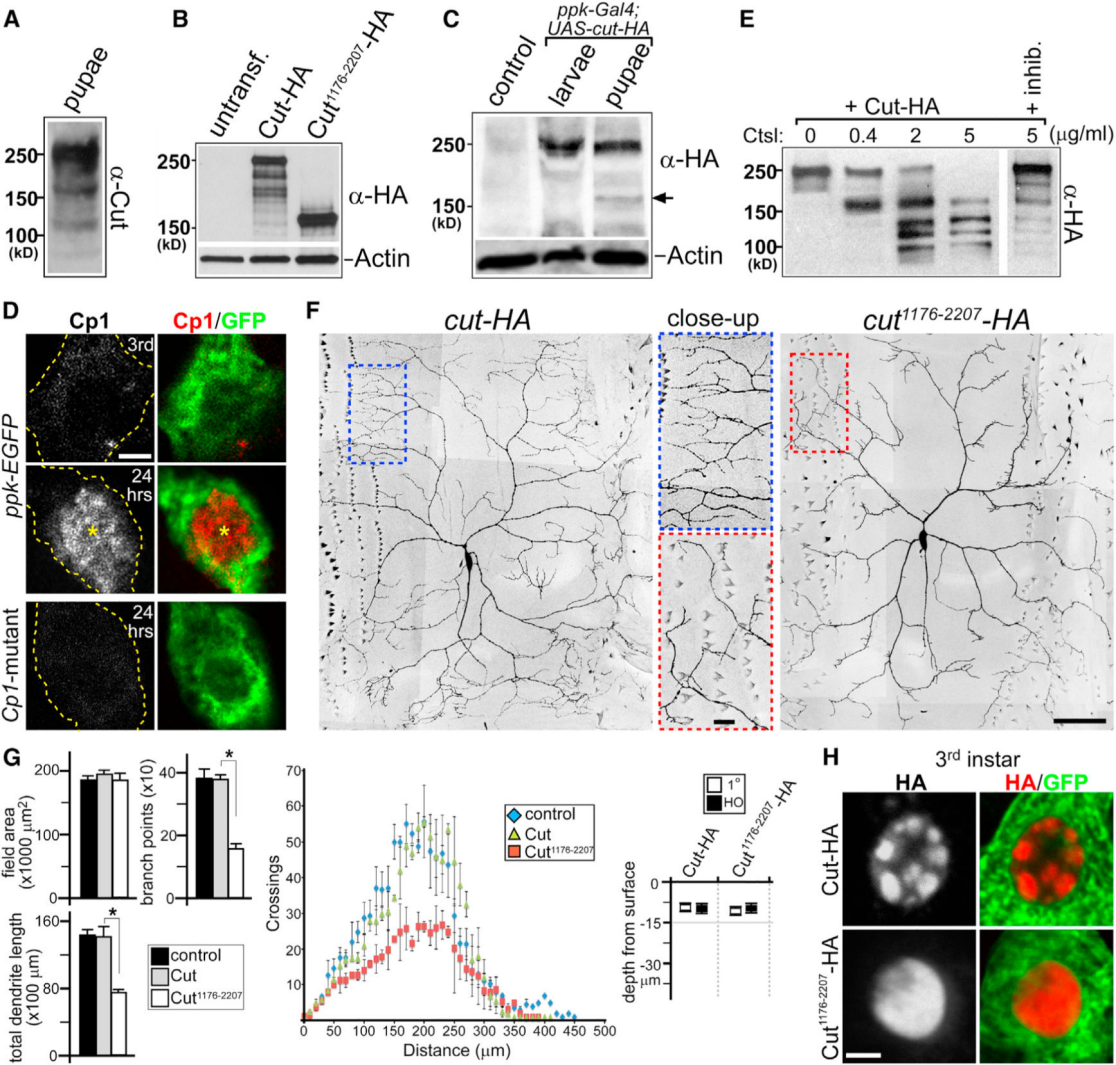
Context-Dependent Production and Function of Truncated Cut Isoform (A–C) Western blot analyses of Cut protein isoforms. (A) Detection of Cut proteins from whole-animal lysate at 24 hr APF. (B) Cut-HA and Cut^1176-2207^-HA protein expression in S2 cells. Note that the major protein bands in each lane, corresponding to intact Cut-HA and Cut^1176-2207^-HA, are near 250 and 160 kDa in size, respectively. untransf., untransfected. (C) Cut-HA expression in *ppk-Gal4; UAS-cut-HA* third-instar larvae and 24 hr APF pupae lysates. Note the appearance of near 160 kDa protein isoform in pupal stage (arrow). Lysate from 24 hr APF *UAS-cut-HA* pupae (no *ppk-Gal4*) is the control. (D) Cp1 plus GFP IHC antibody staining of wild-type ddaC neurons (*ppk-EGFP*) in third-instar larvae (Third), 24 hr APF pupae (24 hr), and *Cp1*-mutant ddaC neuron at 24 hr APF. Each image represents a single confocal plane. Note the nuclear Cp1 staining at 24 hr APF (*) in wild-type ddaC neuron. (E) In vitro cleavage assay using purified Cut-HA protein and Ctsl in increased protease concentrations. Cleavage reaction is sensitive to Ctsl inhibitor Z-FF-FMK (inhib.) (50 μM). (F) Fixed-tissue images of ddaC neurons from third-instar larvae expressing either Cut-HA or Cut^1176-2207^-HA. Color-dashed boxes are shown as corresponding close-up panels. (G) Quantitative analyses of third-instar ddaC neuron dendrite phenotypes: field area, branchpoints, dendrite length, Sholl analysis, and depth from surface (n = 6 in all groups). *p < 0.005, Wilcoxon two-sample test. Error bars represent SEM. (H) Cut-HA and Cut^1176-2207^-HA nuclear staining patterns in third-instar larvae ddaC neurons (*ppk-Gal4; UAS-mCD8::GFP; UAS-cut-HA* or *UAS-cut^1176-2207^-HA*). Scale bars, 2 μm (D and H) and 50 μm (F). See also Figure S4.

To detect Cut protein isoforms in vivo, we performed western blotting analyses on total protein lysates from 24 hr APF pupae. The Cut antibody, recognizing regions surrounding the homeo-domain, identified protein bands about 250, 160, and 110 kDa in size in the 24 hr APF pupae lysate (Figure 4A). To determine which bands corresponded to Cut^1176-2207^, we cotransfected spaghetti squash-Gal4 (sqh-Gal4, expressed in S2 cells) together with either UAS-cut-HA or UAS-cut^1176-2207^-HA DNA constructs into *Drosophila* S2 cells. Western blotting of transfected S2 cell lysates with anti-HA antibody showed that the full-length Cut-HA protein was 250 kDa as predicted, and the truncated Cut^1176-2207^-HA was near 160 kDa in size (Figure 4B). We next made total protein lysates from *ppk-Gal4; UAS-cut-HA* third-instar larvae and 24 hr APF pupae and performed western analyses using anti-HA antibody. This revealed specific induction during metamorphosis of a near 160 kDa Cut protein isoform (Figure 4C). The expression of full-length Cut protein remained robust at 24 hr APF, suggesting that it may contribute to a transcriptional program divergent from truncated Cut protein at this stage.

Generation of truncated mammalian Cux1 protein is thought to involve nuclear localization of Ctsl (Goulet et al., 2004). To determine whether Cp1 is also localized in the nucleus during ddaC neuron dendrite regrowth, we generated a Cp1 antibody. IHC staining comparisons between *ppk-EGFP* third-instar larvae and 24 hr APF pupae showed that whereas levels of Cp1 protein in ddaC neurons are low in larvae, at 24 hr APF, Cp1 expression is greatly upregulated with concentrated nuclear localization (Figure 4D; Supplemental Results). Ctsl activation requires sequential biochemical events that are organelle and pH sensitive, without which the protease remains enzymatically inactive (Collette et al., 2004). Because little is known about Cp1 activation, we performed in vitro cleavage assays using purified active Ctsl and Cut-HA proteins. Sixty-minute incubations of Cut-HA protein in increasing concentrations of Ctsl showed a specific production (at 0.4 μg/ml) of near 160 kDa Cut protein isoform, followed by further cleavages at higher protease concentrations (Figure 4E). These cleavages are sensitive to Ctsl inhibitor Z-FFFMK (Figure 4E).

The stage-dependent changes in Cut protein isoforms raised the possibility that Cut^1176-2207^ functions as a molecular “coincidence detector,” translating hormonally induced signals through Cp1 into lasting dendritic structural changes. This model predicts that activating this program out of context should induce dendritic arbor alterations. Because ecdysone activation induces dendrite pruning first before regrowth (Kuo et al., 2005), and overexpressed Cp1 protein in larval ddaC neurons remained cytoplasmic and gave no obvious phenotype (Figure S3C; data not shown), we tested this possibility of context specificity by expressing Cut^1176-2207^ during dendrite development. Crossing *UAS-cut^1176-2207^-HA* transgene to *ppk-Gal4*; *UAS-mCD8::EGFP* line, third-instar ddaC neurons showed greatly reduced higher-order dendrites as compared to both controls, as well as those expressing full-length Cut-HA (Figures 4F and 4G; data not shown). This third-instar dendrite phenotype was specific to Cut^1176-2207^-HA because we detected no obvious defects when expressing other Cut truncation proteins (Figure S4A; data not shown). Cut^1176-2207^-HA expressed in *cut*-mutant ddaC neurons also resulted in greatly reduced higher-order dendrites in third-instar larvae (Figures S4D and S4E). IHC staining showed that whereas full-length Cut-HA is localized to nuclear spots in third-instar ddaC neurons, Cut^1176-2207^-HA maintained a diffuse nuclear localization at the same stage (Figure 4H).

Our results revealed a surprising mechanism for the same neuron to elaborate distinct dendritic trees after pruning and re-growth. Cp1, as a steroidal hormone-inducible protein, ties extracellular cues to a core transcriptional program first needed for dendrite patterning during early development and modifies it for regrowth. The usage of different Cut isoforms by the ddaC neuron represents an efficient molecular “node” for extracellular information to interact with the developmental program in a context-dependent manner. We have shown that Cp1 is required for normal nuclear localization of Cut transcription factor during ddaC neuron dendrite regrowth after pruning. However, it remains possible that part of this regulation may be Cut cleavage independent. Further experiments to understand the biochemical functions of Cp1, as well as exact DNA binding sites for Cut protein isoforms, will address these questions (also see Supplemental Discussion). Conceptually, it has become increasingly clear that developmental pathways needed to pattern the nervous system are often reused to modulate neuronal plasticity later in life. Revealing these context-dependent usages will be critical for both understanding nervous system function in health and for advancing effective disease treatments.

## EXPERIMENTAL PROCEDURES

### Transgenic Stocks

*UAS-Cp1* was made by standard molecular cloning and DNA injection into embryos. *UAS-cut-HA* and *UAS-cut truncation-HA* lines were made by cDNA insertions into the KpnI site of pUASt-attB and targeted to the attP2 locus via FC31 integrase stocks. Further details are in Supplemental Experimental Procedures.

### Immunohistochemistry

IHC staining was as described (Kuo et al., 2005): chicken anti-GFP (1:2,000; Aves), and mouse anti-HA (1:500; Covance). Cp1 rabbit polyclonal antibody was generated against conjugated peptide FRYIKDNGGIDTEK (by ProSci) and affinity purified (1:20). Larval ddaC neurons were imaged from fixed tissue fillets (1 hr, room temperature) after washing without staining.

### Dendrite Analyses

For depth analyses, dendrites were traced using the Simple Neurite Tracer module in Fiji (http://fiji.sc/Fiji) and measured from the body wall using MATLAB script.

### Biochemistry

*Drosophila* S2 cells were transfected using a 1:4 ratio of sqh-Gal4 to UAS-DNA constructs (1.5 μg DNA/ml media) by a standard calcium phosphate method. Western analyses were performed using a standard protocol: mouse anti-Cut (1:100; Developmental Studies Hybridoma Bank); mouse anti-HA (1:2,000; Covance); rabbit anti-Cp1 (1:50; ProSci); and mouse anti-actin (1:2,000; Abcam). Further details are in Supplemental Experimental Procedures.

## Supplementary Material

Supplementary Figures and Methods
