# Supplementary material for: Cysteine Proteinase-1 and Cut Protein Isoform Control Dendritic Innervation of Two Distinct Sensory Fields by a Single Neuron
Source: Cell Rep. Author manuscript; Available in PMC 2014 Nov 19. (PMC4237277; doi:10.1016/j.celrep.2014.02.003)
Supplement: Supplementary Figures and Methods [file NIHMS618980-supplement-Supplementary_Figures_and_Methods.pdf]

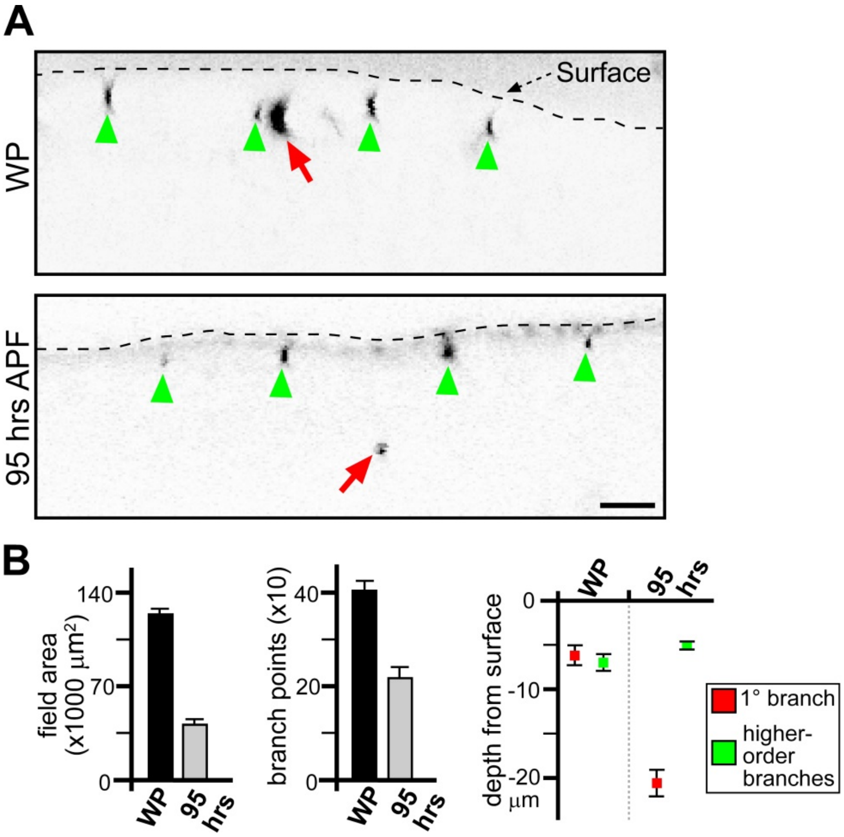

Supplemental Figure 1. Changes in ddaC neuron dendritic arbor architecture following pruning and regeneration during metamorphosis. (A) X-Z cross section views of ddaC neuron dendrites at white pupae (WP) and 95 hrs APF. Primary dendrites are indicated by red arrows, and secondary dendrites by green arrowheads. Note the deeper primary dendritic branch at 95 hrs APF. Dashed line = body wall. Scale bar, 10 μm. (B) Quantitative analyses of ddaC neuron dendritic arbor changes between WP and 95 hrs APF: field area, branch points, depth of primary (1°) and higher-order dendrites from surface. n = 8 in all groups. Error bars represent s.e.m. Images of ddaC neuron dendrites are inverted to black on white for clarity. See also Figure 1.

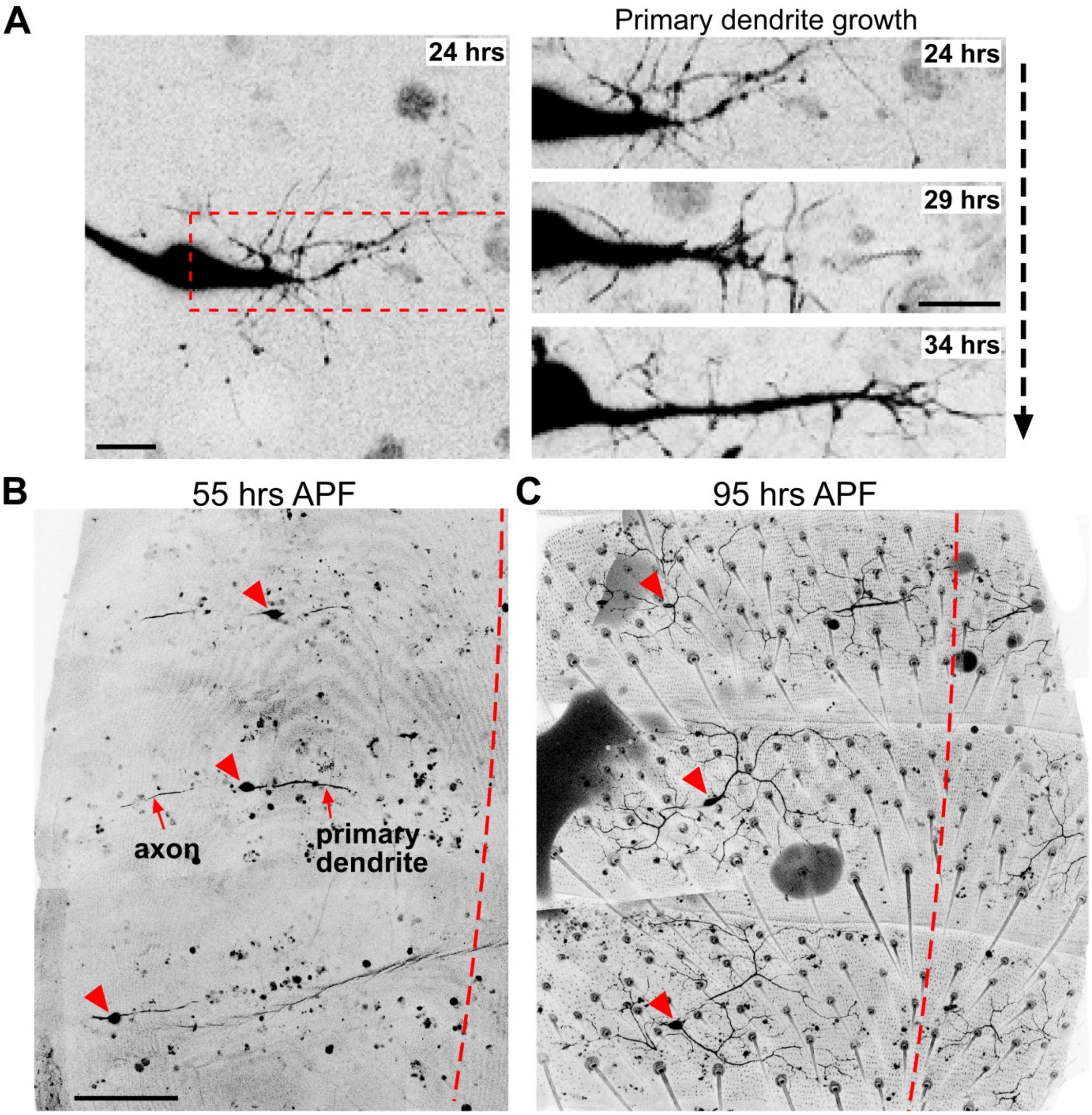

Supplemental Figure 2. Initiation of ddaC neuron dendrite regeneration after pruning during metamorphosis. (A) Representative time-lapse live-imaging of ddaC neuron primary dendrite initiation and growth, at 24, 29, and 34 hrs APF. Red dashed-area in left panel is enlarged in right panel time-lapse series. Note the transient nature of most neurites coming from the primary dendrite at these early time points during generation. See also Movie S2. (B, C) Live imaging large-field collage views of abdominal ddaC neurons during dendrite regeneration at 55 hrs APF (B) and 95 hrs APF (C). Depending on depth from surface, increased laser power is often needed to live-image deep primary dendrites, with corresponding faster rate of surface dendrite photobleaching. Arrowheads point to soma. Dashed lines represent dorsal midline. Scale bars, 10 μm (A), 100 μm (B, C). See also Figure 1.

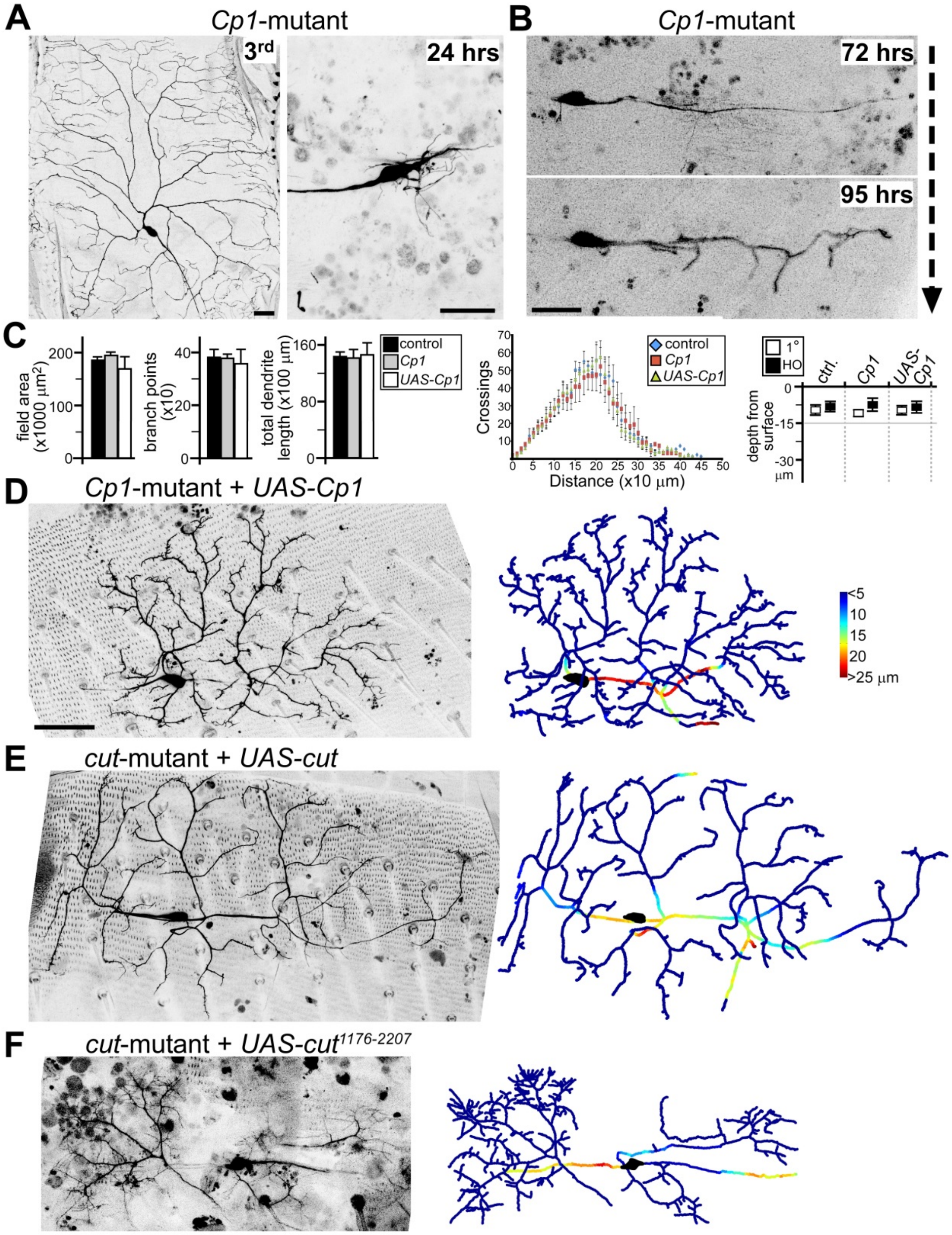

Supplemental Figure 3. (A) *Cp1*-mutant ddaC neuron in 3rd instar larvae (3rd), and at 24 hrs APF after pruning of larval dendrites. (B) Time-lapse live-imaging of *Cp1*-mutant ddaC neuron, imaged first at 72 hrs APF, and again at 95 hrs APF. (C) Quantitative analyses of *Cp1*-mutant and *ppk-Gal4; UAS-Cp1* 3rd instar larval ddaC neuron dendrites: field area, branch points, dendrite length, Sholl analysis, and depth from surface. n = 6 in all groups. (D-F) Live-imaging of ddaC neuron MARCM clones at 95 hrs APF, with corresponding colorimetric representations of dendritic arbor depths in right panels. (D) Representative partial recovery of higher-order dendrites in *Cp1*-mutant clone via *UAS-Cp1* transgene. (E, F) Representative *cut*-mutant clones expressing *UAS-cut* (E) or *UAS-cut*$^{1176-2207}$ (F) transgenes, respectively. Scale bars, 25 μm (A, B), 50 μm (D, E, F). See also Figures 2 and 3.

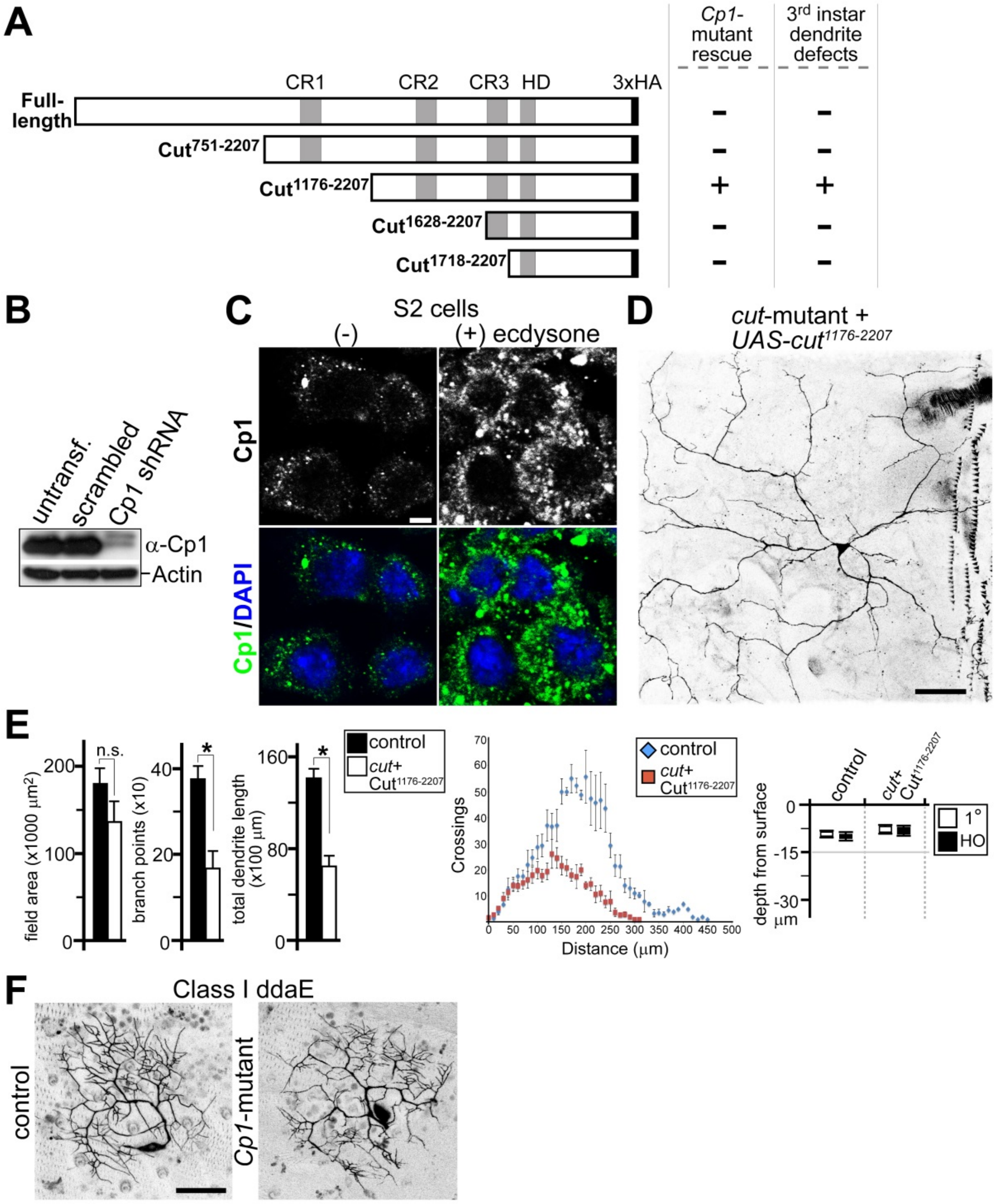

Supplemental Figure 4. (A) Schematic diagram summarizing different HA-tagged Cut protein constructs made into *UAS*-transgenic lines. Their in vivo effects in 1) partial recovery of *Cp1*-mutant dendrite regeneration phenotype during metamorphosis (*Cp1*-mutant rescue), and 2) generating dendritic defects in 3rd instar larvae (3rd instar dendrite defects), are indicated in columns to the right. HD = homeodomain. CR1, 2, 3 = Cut repeats 1, 2, 3. (B) Western blotting with Cp1 antibody on *Drosophila* S2 cell lysates that were either untransfected (untransf.) or transfected with scrambled or *Cp1*-specific shRNA. (C) Cp1 IHC staining on S2 cells with or without ecdysone stimulation. Note the greatly increased cytoplasmic Cp1 expression in ecdysone-treated condition. (D) Representative image of *cut*-mutant ddaC neuron from 3rd instar larvae expressing Cut$^{1176\text{-}2207}$. (E) Quantitative analyses of dendrite phenotypes from (D): field area, branch points, dendrite length, Sholl analysis, and depth from surface. n = 6 in all groups. * P < 0.005, Wilcoxon two-sample test, error bars represent s.e.m. (F) Representative control and *Cp1*-mutant Class I ddaE neuron MARCM clones at 72 hrs APF. Scale bars, 2 μm (C), 50 μm (D, F). See also Figures 3 and 4.

Supplemental Movie Legends

Supplemental Movie 1. This movie shows ddaC neuron dendrite tracings at WP (upper panel) and 95 hours APF (lower panel) rotated in 3 dimensional space. QuickTime (3.8 MB).

Supplemental Movie 2. This movie shows time-lapse live-imaging of a *ppk-EGFP* labeled ddaC neuron regrowing primary dendrite at 35 hours APF. Images were captured once every 5 minutes; total time = 100 minutes. QuickTime (5.5 MB).

Supplemental Movie 3. This movie shows time-lapse live-imaging of a *ppk-EGFP* labeled ddaC neuron elaborating higher-order dendrites from a primary branch, in both X-Y view (upper panel) and X-Z view (lower panel) from 65 to 80 hours APF. Yellow arrowheads point to initiation of higher-order dendrites. Images were captured once every 30 minutes; total time = 15 hours. QuickTime (5.2 MB).

Supplemental Results

Drosophila S2 cells express Cp1 (Kocks et al., 2003) (Figure S4B), and we showed that Cp1 induction in ddaC neurons during metamorphosis is ecdysone-dependent (Figure 2B), consistent with a previous transcriptome study identifying Cp1 as an ecdysone-responsive gene (Chittaranjan et al., 2009). To understand direct Cp1/Cut protein interactions we therefore repeated co-transfection of spaghetti squash-Gal4 together with UAS-cut-HA DNA constructs into S2 cells. While addition of ecdysone to S2 cells significantly enhanced Cp1 expression, the protein remained largely cytoplasmic (Figure S4C) and did not result in full-length Cut protein cleavage in S2 cells (Figure 4B and data not shown). Similarly, *UAS-Cp1* expression in larval ddaC neurons did not result in nuclear-localized Cp1 (data not shown), indicating that Cp1 nuclear localization during metamorphosis is controlled by additional signals. Normally a lysosomal protease, Cp1's mammalian homologue CstI has diverse functions in multiple organ systems including the heart, pancreas, and kidney (Reiser et al., 2010). Thus, it is likely that Cp1 has additional proteolytic substrates/functions important during ddaC neuron dendrite formation and regeneration. Our partial rescue results during the dendrite regrowth phase in *Cp1*-mutant clones showed that Cut protein isoform generation belongs in this pathway.

Supplemental Discussion

Due in large part to technical challenges, the downstream gene targets for Cut needed to pattern dendritic fields during development, and now regrowth, remain unknown. Although we detected some 3rd instar dendrite defects overexpressing Cut using the original *UAS-cut* transgenic stock, similar to previous observations (Jinushi-Nakao et al., 2007) (data not shown), we found no obvious defects using the *UAS-cut-HA* line knocked into the attP2 locus (Figures 4F and 4G), potentially due to Cut expression level differences between the two transgenes. It is likely that mis-expressed Cut$^{1178\text{-}2207}$ in larval ddaC neurons acted out of context to turn on genes normally required for dendrite regrowth after pruning, since the larval and adult sensory fields for these neurons are rather distinct. Mammalian Cux1/Cux2 control dendritic branching of upper layer cortical neurons, through transcriptional regulation of chromatin remodeling genes Xlr3b and Xlr4b (Cubelos et al., 2010). It will be of interest to examine whether similar mechanisms are used by ddaC neurons to remodel dendrites. Furthermore, it will be important to test whether Cux1/Cux2 can be cleaved following extracellular stimuli such as hormonal-induction.

Within the *Drosophila* da neuron family, Class I ddaE neuron also exhibit dendritic arbor structural changes during regrowth after pruning (Williams and Truman, 2004). We did not observe obvious dendrite regrowth phenotypes in *Cp1*-mutant ddaE neurons (Figure S4F), consistent with previous results showing that Cut transcription factor is not required for Class I ddaE neuron dendrite patterning (Grueber et al., 2003a). They raise the intriguing possibility that there may be other protease/transcription factor pairs that can respond to stimuli in a similar fashion, generating structural plasticity in neurons.

Supplemental Experimental Procedures

Fly Stocks

The fly stocks used in this study include: EGFP Flytrap collection (Buszczak et al., 2007; Morin et al., 2001; Quinones-Coello et al., 2007); *Cp1-EGFP* Flytrap line ZCL2854 (Morin et al., 2001); $Cp1^{llcnbw38}$ allele (Gray et al., 1998); $Cp1^{c03987}$ allele (Thibault et al., 2004); FRT^{19A} cut^{c145} MARCM allele and *UAS-cut* (Grueber et al., 2003a); *ppk-EGFP* reporter (Grueber et al., 2003b); *ppk-Gal4* driver (Grueber et al., 2007); *UAS-mCD8::RFP* (gift of E. Gavis); $UAS\text{-}EcR\text{-}DN^{W650A}$ line (Cherbas et al., 2003). Two independent FRT^{42D} recombinant alleles of *Cp1* were generated, followed by MARCM analyses as described (Lee and Luo, 1999).

Live-imaging and Analyses

Images were acquired on Leica SP5 confocal microscope; resonance scanning mode was used for time-lapse experiments. EGFP/mCD8::RFP fluorescence was imaged under identical instrument settings for all time points, with intensities quantified in Fiji.

Biochemistry

For shRNA experiment, S2 cells were first plated for 24 hrs in normal media, then serum was withdrawn for 12 hrs, followed by incubation with 2 μg of dsRNA against Cp1 (cDNA template made by primer pairs 5'-gaaataatacgactcactataggcggtggctcaggccgtttcc-3' and 5'-gaaataatacgactcactataggcagtgtccctgatccttgacggc-3') or generic scrambled dsRNA. Three days after addition of dsRNA cells were harvested for Western analyses. All S2 cell lysates were made by homogenizing in 90°C 5X PAGE buffer (50 mM Tris pH8, 5 mM EDTA, 25% sucrose, 5% SDS, bromophenyl blue), sonicated, and heated at 90°C for 10 minutes. For Cut protein cleavage assay, a poly-6-Histidine tag was added to the N-terminal of full-length Cut-HA protein. His-Cut-HA construct was transfected into HEK293T cells using Lipofectamine 2000. After 48 hr incubation cells were lysed (50 mM Tris-Cl pH 8.0, 300 mM NaCl, 20 mM Imidazole, 1% Triton-X100, protease inhibitor cocktail Roche:11836170001), followed by centrifugation (20K rpm, 30 minutes, 4°C), and supernatant was then coupled to nickel Ni-NTA Agarose resin (Invitrogen: R901-01) for 3 hrs at 4°C. Nickel beads were washed in lysis buffer containing 50 mM Imidazole, and bound His-Cut-HA was eluted from nickel beads in stepwise gradient using 75 mM, 100 mM, 150 mM, 200 mM, and 250 mM Imidazole. Elution fractions containing purified His-Cut-HA were determined through Coomassie-stained SDS PAGE analysis, and confirmed through Western blotting probed with anti-HA antibody. Enzymatically active Cathepsin L (Sigma C6854) and inhibitor Z-FF-FMK (Calbiochem 219421) were used according to manufacturer protocols.

Supplemental References

Buszczak, M., Paterno, S., Lighthouse, D., Bachman, J., Planck, J., Owen, S., Skora, A.D., Nystul, T.G., Ohlstein, B., Allen, A., *et al.* (2007). The carnegie protein trap library: a versatile tool for Drosophila developmental studies. Genetics *175*, 1505-1531.

Cherbas, L., Hu, X., Zhimulev, I., Belyaeva, E., and Cherbas, P. (2003). EcR isoforms in Drosophila: testing tissue-specific requirements by targeted blockade and rescue. Development *130*, 271-284.

Chittaranjan, S., McConechy, M., Hou, Y.C., Freeman, J.D., Devorkin, L., and Gorski, S.M. (2009). Steroid hormone control of cell death and cell survival: molecular insights using RNAi. PLoS Genet. *5*, e1000379.

Cubelos, B., Sebastian-Serrano, A., Beccari, L., Calcagnotto, M.E., Cisneros, E., Kim, S., Dopazo, A., Alvarez-Dolado, M., Redondo, J.M., Bovolenta, P., *et al.* (2010). Cux1 and Cux2 regulate dendritic branching, spine morphology, and synapses of the upper layer neurons of the cortex. Neuron *66*, 523-535.

Gray, Y.H., Sved, J.A., Preston, C.R., and Engels, W.R. (1998). Structure and associated mutational effects of the cysteine proteinase (CP1) gene of Drosophila melanogaster. Ins. Mol. Biol. *7*, 291-293.

Grueber, W.B., Jan, L.Y., and Jan, Y.N. (2003a). Different levels of the homeodomain protein cut regulate distinct dendrite branching patterns of Drosophila multidendritic neurons. Cell *112*, 805-818.

Grueber, W.B., Ye, B., Moore, A.W., Jan, L.Y., and Jan, Y.N. (2003b). Dendrites of distinct classes of Drosophila sensory neurons show different capacities for homotypic repulsion. Curr. Biol. *13*, 618-626.

Grueber, W.B., Ye, B., Yang, C.H., Younger, S., Borden, K., Jan, L.Y., and Jan, Y.N. (2007). Projections of Drosophila multidendritic neurons in the central nervous system: links with peripheral dendrite morphology. Development *134*, 55-64.

Jinushi-Nakao, S., Arvind, R., Amikura, R., Kinameri, E., Liu, A.W., and Moore, A.W. (2007). Knot/Collier and cut control different aspects of dendrite cytoskeleton and synergize to define final arbor shape. Neuron *56*, 963-978.

Kocks, C., Maehr, R., Overkleeft, H.S., Wang, E.W., Iyer, L.K., Lennon-Dumenil, A.M., Ploegh, H.L., and Kessler, B.M. (2003). Functional proteomics of the active cysteine protease content in Drosophila S2 cells. Mol. Cell. Proteomics *2*, 1188-1197.

Lee, T., and Luo, L. (1999). Mosaic analysis with a repressible cell marker for studies of gene function in neuronal morphogenesis. Neuron *22*, 451-461.

Morin, X., Daneman, R., Zavortink, M., and Chia, W. (2001). A protein trap strategy to detect GFP-tagged proteins expressed from their endogenous loci in Drosophila. Proc. Natl. Acad. Sci. USA *98*, 15050-15055.

Quinones-Coello, A.T., Petrella, L.N., Ayers, K., Melillo, A., Mazzalupo, S., Hudson, A.M., Wang, S., Castiblanco, C., Buszczak, M., Hoskins, R.A., and Cooley, L. (2007). Exploring strategies for protein trapping in Drosophila. Genetics *175*, 1089-1104.

Reiser, J., Adair, B., and Reinheckel, T. (2010). Specialized roles for cysteine cathepsins in health and disease. J. Clin. Inv. *120*, 3421-3431.

Thibault, S.T., Singer, M.A., Miyazaki, W.Y., Milash, B., Dompe, N.A., Singh, C.M., Buchholz, R., Demsky, M., Fawcett, R., Francis-Lang, H.L., *et al.* (2004). A complementary transposon tool kit for Drosophila melanogaster using P and piggyBac. Nat. Genet. *36*, 283-287.

Williams, D.W., and Truman, J.W. (2004). Mechanisms of dendritic elaboration of sensory neurons in Drosophila: insights from in vivo time lapse. J. Neurosci. *24*, 1541-1550.
